# Association Between Coffee Consumption and Metabolic Syndrome Components Among Saudi Adults

**DOI:** 10.3390/metabo15030163

**Published:** 2025-03-01

**Authors:** Wala I. Alzahrani, Sarah N. Alsharif, Maryam S. Hafiz, Doaa A. Alyoubi, Amal M. Alrizqi, Raneem A. Younes, Alaa M. Jahlan, Khaled A. Yaghmour

**Affiliations:** 1 Department of Clinical Nutrition, Faculty of Applied Medical Sciences, King Abdulaziz University, Jeddah 21589, Saudi Arabia; snalsharif@kau.edu.sa (S.N.A.); mshafiz@kau.edu.sa (M.S.H.); daalyoubi@kau.edu.sa (D.A.A.); adaifallahalrizqi@stu.kau.edu.sa (A.M.A.); rayounes@stu.kau.edu.sa (R.A.Y.); aomarjahlan@stu.kau.edu.sa (A.M.J.); 2 Family Medicine Department, Faculty of Medicine, King Abdulaziz University, Jeddah 21589, Saudi Arabia; kyaghmour@kau.edu.sa

**Keywords:** association, coffee, consumption, MetS, components, adults

## Abstract

Background: Previous research has linked high coffee consumption to an increased risk of metabolic syndrome (MetS). This study aimed to assess the relationship between coffee consumption and MetS components among Saudi adults. Methods: A cross-sectional study was performed on adults who met at least three criteria for a MetS diagnosis. Data concerning demographics, lifestyle, sleeping patterns, medical health, anthropometric measurements, habitual coffee drinking, and lab levels for HDL-C, LDL-C, TC, TGs, HbA1c, and FBG were collected. Results: Of the 95 participants, 51% were women, 75.8% were >50 years old, 75.8% were obese, 62% were used to practicing physical activity, 74.5% never smoked, 56.4% slept < 7 h/day, and 89.5% were coffee consumers. Of these, 94.7% had high waist circumference, 63.2% had high BP, 47.4% had high FBG, 41.1% had low HDL, and 23.2% had high TGs. For coffee consumers, 37.6% drank a small cup, 34.5% drank coffee once daily, 89.4% drank Arabic coffee, and 75.3% added no additives. Conclusions: No significant association was found between coffee consumption patterns and any MetS component, with the exception of elevated TGs, which was strongly associated with coffee cup size and number of daily cups. Waist circumference and BMI had a strong positive correlation with coffee cup size, and there was a significant relationship between the number of daily cups, BMI, and TC. Further prospective studies are needed to establish a causal relationship.

## 1. Introduction

Metabolic syndrome (MetS) is defined as the existence of many recognized risk factors for cardiovascular disease, including insulin resistance, obesity, dyslipidemia, and hypertension [1]. In Saudi Arabia, in 2018, the prevalence of epidemic MetS was 39.8% (34.4% in men and 29.2% in women), with higher rates in men and the elderly [2].

Coffee is the world’s second most popular beverage after water. It is enjoyed for its aromatic flavors and delicious taste, with around 500 billion cups consumed each year [3]. According to research by the Saudi Press Agency (SPA), Saudi Arabia is one of the top ten countries for coffee consumption. The nation imports 70,000 to 90,000 tons of coffee annually, with an annual expenditure of more than SAR 1 billion for this beverage [4].

According to the Institute for Coffee Studies, coffee contains more than 1500 chemical components. The primary water-soluble components are phenolic polymers (8%), polysaccharides (6%), chlorogenic acids (4%), minerals (3%), water (2%), caffeine (1%), organic acids (0.5%), sugars (0.3%), lipids (0.2%), and fragrance (0.1%). While coffee was long thought to be dangerous due to the hazards of caffeine dependence, new research has revealed that it may have metabolic benefits due to its polyphenol components, which have antioxidant and anti-inflammatory characteristics [3]. These potential benefits include a lower risk of Alzheimer’s disease, type 2 diabetes (T2DM), Parkinson’s disease, and certain types of cancer and an increased metabolic rate. Coffee consumption has been related to potential benefits in terms of T2DM, with various studies indicating a risk reduction with varying amounts of intake. Research suggests that drinking a lot of coffee can boost insulin production, sensitivity, and β-cell function.

In terms of the impact of coffee consumption on waist circumference (WC) and body weight, the precise methods by which coffee components influence body composition remain unclear. Caffeine, a significant component of coffee, has been shown to increase lipolytic activity, cellular thermogenesis, norepinephrine release, and adrenaline hormone activity by acting as an adenosine receptor antagonist [5]. In an earlier research study, the impact of extended coffee consumption on 1141 individuals over 7.6 years revealed a notable decrease in WC with higher coffee intake levels [6].

While acute coffee consumption has been shown to momentarily elevate blood pressure (BP), the effects of long-term use are not entirely understood. Chronic coffee consumption may not have a long-term impact on BP. Furthermore, meta-analyses have shown that the regular consumption of more than three cups of coffee per day is not connected with hypertension (HTN). Caffeine, a coffee component, is thought to impact heart rate and BP via a variety of physiological routes [5]. A 6-year study investigated the impact of consistent coffee intake on BP levels and discovered a link between regular coffee consumption and lower BP [7]. In a different study focusing on the influence of Arabic coffee on persons with stage 1 HTN, Arabic coffee considerably lowered both systolic and diastolic BP levels [8].

When evaluating the effects of coffee on blood lipids, it is worth noting that the amounts of Cafestol and Kahweol, two chemicals in coffee linked to elevated cholesterol, can be largely reduced by using a paper filter. However, prolonged cooking can increase these cholesterol-raising chemicals [5]. A previous study found that drinking coffee was connected to lower levels of blood triglycerides (TGs) [9]. A different study on the relationship between coffee and lipid metabolism showed that consistent filtered coffee consumption over three months had a favorable impact on high-density lipoprotein cholesterol (HDL-C) levels [10].

Previous studies suggest that moderate coffee drinking might favorably influence clinical cardiovascular risk factors [11]. One study found that drinking more than one cup of coffee per day could lower the prevalence of MetS in Taiwanese men and women [12]. However, a separate study conducted in Korea for two years revealed no significant link between coffee drinking and MetS [13]; furthermore, other researchers have shown that coffee intake among Korean women is associated with a higher frequency of obesity [14].

There is minimal information for the Saudi population regarding the relationship between coffee consumption and MetS components. A cross-sectional study conducted in Saudi Arabia on healthy overweight and obese participants investigated the connection between dietary caffeine consumption and MetS components. Higher dietary caffeine intake was found to be associated with decreased fat mass, increased fat-free mass, and lower total cholesterol (TC) and low-density lipoprotein cholesterol (LDL-C) values. Furthermore, greater dietary coffee intake was associated with lower insulin resistance and higher HDL-C levels in the Saudi population [15]. These inconsistent results could be attributed to varying research methodologies and a lack of detailed knowledge about different types of coffee. Furthermore, in Saudi Arabia, studies are being undertaken on coffee consumption and MetS risk factors, but not on MetS components [16,17]. Hence, this study aimed to assess the relationship between coffee consumption and MetS components among Saudi adults at King Abdulaziz University Hospital (KAUH).

## 2. Materials and Methods

### 2.1. Study Population and Design

This cross-sectional study was approved by the Biomedical Ethics Research Committee at King Abdulaziz University in Jeddah, Saudi Arabia (No. 619-23). A total of 156 participants were recruited voluntarily, with oral consent obtained prior to their participation. Data collection was conducted over three months, and participants were selected from KAUH. The inclusion criteria were Saudi adults aged 30 and above, both men and women, who met at least three criteria for a MetS diagnosis. Participants who completed anthropometric measurements, a habitual coffee drinking questionnaire, and a two-day, 24 h food recall and had relevant lab values in their medical records were included. The exclusion criteria involved participants of other nationalities; those with insufficient data for a MetS diagnosis, such as blood lipid profiles or blood glucose levels; and women who were pregnant or breastfeeding. Finally, 95 participants were eligible for statistical analysis. The sample size was calculated using G Power 3.1.9.7, with a total of approximately 90 participants. Data were collected from participants through a self-administered questionnaire and face-to-face interviews.

### 2.2. Anthropometric Measurements and Definition of MetS

Nurses from KAUH conducted anthropometric measurements, recording weight (kg) and height (cm), while a registered dietitian measured WC in the screening room. Height and weight were measured using a Seca stadiometer scale. These measurements were typically taken once unless inconsistencies were observed. BP was measured using a SureSigns VS4 vital signs monitor after participants had rested in a seated position for at least five minutes. Two readings were taken at 1–2 min intervals, and the average of the two closest values was recorded. WC was measured with a non-stretchable measuring tape at the midpoint between the lower rib and iliac crest, with two measurements taken and averaged as the final value.

Additionally, body mass index (BMI) was calculated, and weight status was evaluated using the following WHO cut-off points: underweight (<18.5 kg/m^2^), normal (≥18.5 and <24.9 kg/m^2^), overweight (≥25 and <29.9 kg/m^2^), and obese (≥30 kg/m^2^), including obese class I (30 to 34.9 kg/m^2^), obese class II (35 to 39.9 kg/m^2^), and obese class III (≥40 kg/m^2^) [18,19]. Biochemical measurements, including HDL-C, LDL-C, TC, TGs, and hemoglobin A1c (HbA1c), were collected from KAUH medical records. MetS was identified based on the Harmonizing Definition established by the International Diabetes Federation (IDF), the American Heart Association, and the National Heart, Lung, and Blood Institute (AHA/NHLBI) if at least three of the following five metabolic abnormalities were present: (1) WC of ≥94 cm for men and ≥80 cm for women, as defined for the Middle East population until new data are available; (2) TGs of ≥150 mg/dL or on drug treatment for elevated TGs; (3) HDL-C level of <40 mg/dL in men or <50 mg/dL in women or on drug treatment for reduced HDL-C; (4) systolic BP of ≥130 mmHg or diastolic BP of ≥85 mmHg or on antihypertensive drug treatment in a patient with a history of HTN; (5) fasting blood glucose (FBG) level of ≥100 mg/dL or on drug treatment for elevated FBG [20,21].

Dietary intake was assessed using a two-day, 24 h recall. A previously validated habitual caffeine consumption questionnaire was combined with a two-day, 24 h recall to assess coffee consumption [22]. This questionnaire covered the weekly volume intake of coffee, which ranged from small cups (273 mL) to extra-large cups (591 mL). Participants were asked to estimate the average number of cups consumed according to four categories: 1, 2, 3, and >3 cups per day. Participants were also asked about their preferred coffee types (Arabic, Turkish, black, cappuccino, latte, instant, and others) and any additives they typically use, such as milk, cream, sugar, and sweeteners. Black coffee was described as coffee powder or extracts without other ingredients.

### 2.3. Assessment of Other Covariates

Through a questionnaire, we obtained demographic data, such as age, gender, nationality, educational level, marital status, occupational status, and socioeconomic status. The lifestyle characteristics of the participants were also documented, including their smoking habits, both direct and indirect exposure, and categories of physical activity levels ranging from never to >5 times per week. Sleeping patterns were categorized into less than 7 h per day, 7–8 h per day, and more than 8 h per day. Medical health data encompassed the frequency of taking multivitamins or minerals, medications, family history, and the presence of medical conditions, which were categorized as having two or more, one, or none of the following: cancer, diabetes, heart disease, depression, obesity, liver disease, high cholesterol, and high BP.

### 2.4. Data Analysis

Data collected were tabulated and analyzed using SPSS (Statistical Package for Social Science) version 26.0 on an IBM-compatible computer. Two types of statistical analysis were performed: (1) descriptive statistics, e.g., number (No), percentage (%), qualitative data, and mean ± SD for quantitative data; (2) analytic statistics, which included the following tests: Student’s *t*-test, a parametric test used to compare two quantitative variables; the Chi-squared test (χ^2^), a parametric test used to find associations between two or more qualitative variables; and the Spearman correlation test, a parametric test used to find the possible correlation between two quantitative variables. Additionally, multivariate logistic regression analysis was performed to explore the relationship between coffee consumption and MetS components. A *p*-value of <0.05 was considered statistically significant.

## 3. Results

**Figure 1:**
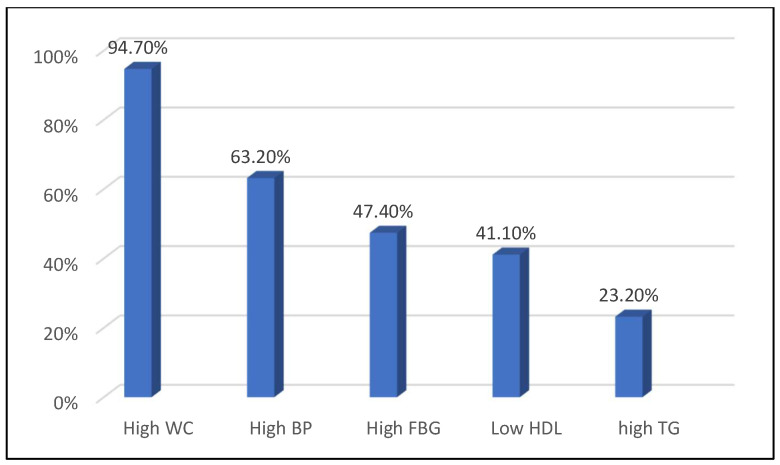
A bar chart displaying the rate of metabolic syndrome components among the participants (N = 95).

Regarding the 95 participants, the sociodemographic data show that 53.7% were women and 46.3% were men. Regarding age distribution, 75.8% were over 50 years, 13.7% were between 36 and 40 years, 7.4% were between 46 and 49 years, 2.1% were between 30 and 35 years, and 1.1% were between 41 and 45 years. Most participants (80%) were married, while the remaining were divorced, single, or widowed. Regarding education status, 34.7% held a master’s degree. Interestingly, 42.1% were unemployed, 32.6% were retired, and only 25.3% were employed. In terms of monthly income, 45.2% of participants earned more than SAR 10,000. In terms of BMI, 75.8% were classified as obese, 15.8% as overweight, and 8.4% had a normal BMI. The lifestyle characteristics data show that 62% were used to practicing physical activity, 74.5% had never smoked, and 56.4% slept < 7 h/day. Regarding coffee consumption, 89.5% were coffee consumers. Regarding the components of MetS, 94.7% had high WC, 63.2% had high BP, 47.4% had high FBG, 41.1% had low HDL, and 23.2% had high TGs (Figure 1).

Regarding coffee consumption characteristics among the 85 participants, 37.6% consumed coffee in a small cup (237 mL), 24.7% consumed coffee in a medium cup (350 mL), 14.1% consumed coffee in a large cup (473 mL), and 23.5% consumed coffee in an XL cup (591 mL). In terms of daily consumption, 34.5% drank coffee once, 19% twice, 19% three times, 20.2% more than three times, and 7.1% only once a week. The majority (89.4%) consumed Arabic coffee, followed by 31.8% who consumed black coffee, 15.3% who consumed cappuccino or instant coffee, 10.6% who consumed Turkish coffee, 2.4% who consumed mochas, and 1.2% who consumed Americano, cold coffee, or lattes. Regarding additives, 75.3% consumed coffee with no additives, while 15.3% added milk, 10.6% added sugar, 3.5% used sweeteners, 2.4% used stevia sugar, and 1.2% added syrup or shredded chocolate.

Table 1 shows no significant difference between coffee consumers and non-consumers regarding any component of MetS except for low HDL, as the rate of low HDL was significantly higher among coffee consumers (*p* = 0.035).

Table 2 shows no significant relationship between coffee consumption characteristics and any component of MetS among the 85 participants except for high TGs, which showed a significant relationship with both coffee cup size and the number of cups consumed daily (*p* = 0.022 and 0.013, respectively).

**Figure 2:**
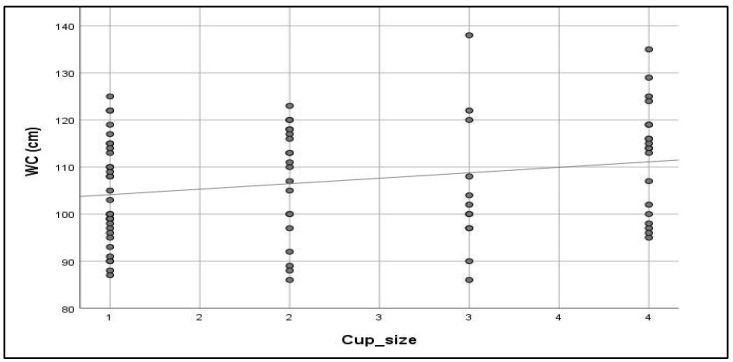
A scatter plot demonstrating the correlation between coffee cup size and WC (N = 85).

At the same time, Table 3 and Figure 2 show no significant correlation between coffee consumption characteristics and any MetS parameter except for WC, which showed a significant positive correlation with coffee cup size (*p* = 0.038).

Table 4 shows no significant relationship between coffee consumption characteristics and BMI, TC, or LDL except for the number of cups consumed daily, which showed a significant relationship with both BMI and TC (*p* = 0.008 and 0.046, respectively).

At the same time, Table 5 shows no significant correlation between coffee consumption characteristics and BMI, TC, or LDL except for BMI, which showed a significant positive correlation with coffee cup size (*p* = 0.009).

A logistic regression analysis was performed to assess whether coffee consumption was a risk factor (independent predictor) for any MetS components. There were no significant predictors of high WC, high TGs, low HDL, or high BP in the coffee consumption data. Table 6 shows that consuming coffee without additives is a significant protector against high FBG according to the coffee consumption data.

## 4. Discussion

MetS is thought to affect 39.8% of the Saudi population, with higher rates among men and the elderly [2]. In contrast, our findings demonstrated a higher prevalence of MetS among women (51%) and elderly patients over the age of 50 (75.8%). Previous studies have shown that coffee consumption protects against HTN, reduces MetS components, and lowers MetS risk and severity by lowering BP, TG levels, and BMI [12,23,24]. As a result, the goal of this study was to investigate the association between coffee consumption and MetS components in 95 individuals from KAUH, Saudi Arabia.

The current study showed that coffee consumption has a considerable negative effect on HDL-C levels. A study published in the European Journal of Epidemiology found that moderate and high coffee drinking was substantially linked with decreased HDL-C levels in women [25]. These findings contradict a study conducted among Taiwanese adults, which showed that coffee drinking was associated with higher levels of HDL-C, implying that coffee consumption may have a favorable or natural influence on HDL-C or MetS components [26]. This was explained in a previous study on coffee’s antiatherogenic properties, which increase the expression of the ATP-binding cassette transporter (ABCG1) and scavenger receptor class B type I (SR-BI) and enhance HDL-mediated cholesterol efflux from macrophages via plasma phenolic acids [27].

In contrast, Panagiotakos et al. found that consuming coffee had no significant influence on HDL-C levels. The study comprised 937 elderly people aged 65 to 100 years [28]. Miranda et al. found that coffee consumption had no significant influence on HDL-C levels in 557 healthy volunteers from São Paulo, Brazil [11]. However, the current investigation found no statistically significant differences between coffee and non-coffee consumers in terms of MetS components. Baulk et al. reported that coffee consumption was not linked to any metabolic syndrome components [25].

Furthermore, we found a considerable positive association between WC and coffee cup size. This finding is consistent with prior research investigating the influence of coffee consumption on adiposity [13,14,29]. However, no significant associations were discovered between coffee consumption characteristics and other MetS components, with the exception of high TG levels, which were strongly linked to both coffee cup size and number of cups consumed each day. A prior study found that coffee consumption increased TG levels [30].

Furthermore, an RCT meta-analysis found a dose–response connection between coffee consumption and TG levels [25]. In contrast, a recent study conducted in Japan revealed that increased coffee consumption lowered the risk of elevated TG levels but not WC or BP [9]. However, other research has shown contradictory findings. A cross-sectional study conducted in a Western population found no significant association between coffee consumption and TG levels, which could be attributed to coffee preparation methods, genetic predispositions, or dietary habits [31].

The strong associations between coffee consumption and increased TG and WC levels imply that coffee may have a dose-dependent or quantity-related effect on lipid metabolism. These findings may indicate that specific coffee consumption patterns aggravate dyslipidemia in individuals, particularly those with high TG levels and WC, which is a crucial component of MetS. However, the absence of substantial associations with other MetS components shows that coffee’s impact on metabolic health may be selective rather than universally damaging or beneficial to all metabolic parameters.

Several studies have identified a significant link between coffee consumption and BMI. Tabrizi, R. et al. (2018) conducted a systematic review and meta-analysis of RCTs and found that coffee consumption promotes BMI reduction [32]. Other research has not demonstrated a link between coffee consumption and BMI. A large cohort study conducted by Freedman et al. (2012) found no relationship between coffee consumption and BMI across age groups [33]. These inequalities could be due to differences in food, physical activity, and genetics among the study participants.

The current study demonstrated a strong relationship between the number of coffee cups consumed each day and both BMI and TC. Another study found that drinking espresso coffee was associated with higher total blood cholesterol levels [34]. At the same time, another study found that excessive coffee drinking was associated with elevated LDL cholesterol levels [30]. However, that study’s lack of a correlation between coffee intake characteristics and LDL-C suggests that factors such as coffee preparation methods, individual genetics, and lifestyle differences may play important roles.

The current investigation demonstrated no relationship between coffee additives and MetS components, with the exception of consuming coffee without additives, which was associated with increased FBG. In contrast, a previous study found that adding sugar or artificial sweeteners significantly reduced the inverse relationship between increasing coffee consumption and T2DM risk, whereas cream had no influence on this relationship [35]. Tan et al., 2021, showed that consumers of three-in-one coffee (coffee with sugar and creamer) were less likely to have low HDL-C and high FPG [36].

Our logistic regression showed that coffee intake data were not a significant predictor of high WC, high TGs, low HDL, or high blood pressure. This finding contrasts with that of Stutz et al., who found that moderate and high coffee consumption was related to an elevated risk of metabolic syndrome. Furthermore, any degree of coffee consumption was linked to an elevated risk of the blood pressure component [37]. Baspinar et al. observed that among the metabolic syndrome components, the risk of type 2 diabetes decreased by 7.0% with each cup of coffee drunk daily. Coffee’s components are hypothesized to have antidiabetic effects by boosting insulin sensitivity and release, enhancing β-cell activity, and blocking glucose transporters [5].

The contradictory results of previous studies necessitate longitudinal studies to further assess the association between coffee consumption patterns and metabolic syndrome components.

### 4.1. Study Strengths

This was the first study performed in Saudi Arabia to assess the relationship between coffee consumption and MetS components. A large sample of both genders was included in this cross-sectional study to provide stronger evidence. We assessed various coffee consumption patterns, such as coffee cup size, number of coffee cups per day, types of coffee, and coffee additives.

### 4.2. Limitations

As the study design was cross-sectional, we could not define a causal relationship between coffee consumption and MetS components. Another limitation was our hypothesis that the amount of coffee consumed would impact the anthropometric measurements of the subjects; however, the potential influence of confounding variables, such as nutrition, on these outcomes was not assessed.

## 5. Conclusions

This study observed no significant difference between coffee consumers and non-consumers regarding any component of MetS, except for the rate of low HDL, which was significantly higher among coffee consumers. In addition, no significant relationship was found between coffee consumption characteristics and any MetS component except for high TGs, which was significantly associated with both coffee cup size and the number of cups consumed daily. WC and BMI had a significant positive correlation with coffee cup size, and a significant association was found between the number of daily cups and both BMI and TC. Despite the potential health effects of coffee consumption on low HDL, high TGs, WC, and BMI, we recommend future longitudinal studies and well-designed randomized controlled trials to confirm the causative relationship.

## Figures and Tables

**Table 1:** Relationship between coffee consumption and MetS components (N = 95).

		Coffee Consumption		
Variable	Categories	Yes (N = 85)	No (N = 10)	*p*-Value *
High WC	No	4 (4.7%)	1 (10%)	0.478
	Yes	81 (95.3%)	9 (90%)	
High BP	No	30 (35.3%)	5 (50%)	0.362
	Yes	55 (64.7%)	5 (50%)	
High FBG	No	45 (52.9%)	5 (50%)	0.860
	Yes	40 (47.1%)	5 (50%)	
Low HDL	No	47 (55.3%)	9 (90%)	0.035
	Yes	38 (44.7%)	1 (10%)	
High TGs	No	64 (75.3%)	9 (90%)	0.297
	Yes	21 (24.7%)	1 (10%)	

* Chi-squared test.

**Table 2:** Relationship between coffee consumption and MetS components (N = 85).

		High WC			High BP			High TGs			Low HDL			High FBG		
		Yes (n = 81)	No (n = 4)	*p*-Value	Yes (n = 55)	No (n = 30)	*p*-Value	Yes (n = 21)	No (n = 64)	*p*-Value	Yes (n = 38)	No (n = 47)	*p*-Value	Yes (n = 40)	No (n = 45)	*p*-Value
Coffee cup size	S	31 (38.3%)	1 (25%)	0.602	23 (41.8%)	9 (30%)	0.243	10 (47.6%)	22 (34.4%)	0.022	13 (34.2%)	19 (40.4%)	0.488	15 (37.5%)	17 (37.8%)	0.702
	M	19 (23.5%)	2 (50%)		11 (20%)	10 (33.3%)		1 (4.8%)	20 (31.3%)		12 (31.6%)	9 (19.1%)		10 (25%)	11 (24.4%)	
	L	12 (14.8%)	0		6 (10.9%)	6 (20%)		6 (28.6%)	6 (9.4%)		6 (15.8%)	6 (12.8%)		4 (10%)	8 (17.8%)	
	XL	19 (23.5%)	1 (25%)		15 (27.3%)	5 (16.7%)		4 (19%)	16 (25%)		7 (13.4%)	13 (27.7%)		11 (27.5%)	9 (20%)	
Coffee cups/day	<1 (once a week)	6 (7.4%)	0	0.429	4 (7.3%)	2 (6.7%)	0.373	2 (7.4%)	4 (6.3%)	0.013	2 (5.3%)	4 (8.5%)	0.605	1 (2.5%)	5 (11.1%)	0.491
	1	30 (37%)	0		18 (32.7%)	12 (40%)		3 (37%)	27 (42.2%)		12 (31.6%)	18 (38.3%)		15 (37.5%)	15 (33.3%)	
	2	15 (18.5%)	1 (25%)		8 (14.5%)	8 (26.7%)		9 (18.5%)	7 (10.9%)		10 (26.3%)	6 (12.8%)		7 (17.5%)	9 (20%)	
	3	15 (18.5%)	1 (25%)		11 (20%)	5 (31.3%)		3 (18.5%)	13 (20.3%)		7 (18.4%)	9 (19.1%)		7 (17.5%)	9 (20%)	
	> 3	15 (18.5%)	2 (50%)		14 (25.5%)	3 (10%)		4 (18.5%)	13 (20.3%)		7 (18.4%)	10 (21.3%)		10 (25%)	7 (15.6%)	
Types of coffee	Arabic	73 (90.1%)	3 (75%)	0.243	48 (87.3%)	28 (93.3%)	0.822	21 (100%)	55 (85.9%)	0.496	34 (89.5%)	42 (89.4%)	0.132	28 (84.8%)	48 (92.3%)	0.522
	Cappuccino	12 (14.8%)	1 (25%)		8 (14.5%)	5 (16.7%)		4 (19%)	9 (14.1%)		6 (15.8%)	7 (14.9%)		4 (12.1%)	9 (17.3%)	
	Black	25 (30.9%)	2 (50%)		20 (36.4%)	7 (23.3%)		7 (33.3%)	20 (31.3%)		13 (34.2%)	14 (29.8%)		14 (42.4%)	13 (25%)	
	Turkish	8 (9.9%)	1 (25%)		7 (12.7%)	2 (6.7%)		2 (9.5%)	7 (10.9%)		4 (10.5%)	5 (10.6%)		3 (9.1%)	6 (11.5%)	
	Instant	13 (16%)	0		10 (18.2%)	3 (10%)		0	13 (20.3%)		6 (15.8%)	7 (14.9%)		3 (9.1%)	10 (19.2%)	
	Americano	1 (1.2%)	0		1 (1.8%)	0		0	1 (1.6%)		1 (2.6%)	0		1 (3.0%)	0	
	Cold	1 (1.2%)	0		1 (1.8%)	0		0	1 (1.6%)		1 (2.6%)	0		1 (3.0%)	0	
	Latte	1 (1.2%)	0		1 (1.8%)	0		1 (4.8%)	0		1 (2.6%)	0		0	1 (1.9%)	
	Mocha	2 (2.5%)	0		1 (1.8%)	1 (3.3%)		0	2 (3.1%)		1 (2.6%)	1 (2.1%)		1 (3.0%)	1 (1.9%)	
Coffee additives	Milk	13 (16%)	0	0.921	11 (20%)	2 (6.7%)	0.369	5 (23.8%)	8 (12.5%)	0.585	7 (18.4%)	6 (12.8%)	0.137	2 (6.1%)	11 (21.2%)	0.368
	Sugar	9 (11.1%)	0		6 (10.9%)	3 (10%)		3 (14.3%)	6 (9.4%)		5 (13.2%)	4 (8.5%)		0	9 (17.3%)	
	Sweetener	3 (3.7%)	0		3 (5.5%)	0		1 (4.8%)	2 (3.1%)		0	3 (6.4%)		1 (3.0%)	2 (3.8%)	
	Syrup	1 (1.2%)	0		1 (1.8%)	0		1 (4.8%)	0		0	1 (2.1%)		0	1 (1.9%)	
	Shredded chocolate	1 (1.2%)	0		0	1 (3.3%)		0	1 (1.6%)		0	1 (2.1%)		0	1 (1.9%)	
	Stevia sugar	2 (2.5%)	0		0	2 (6.7%)		0	2 (3.1%)		0	2 (4.3%)		1 (3.0%)	1 (1.9%)	
	None	60 (64.1%)	4 (100%)		40 (72.7%)	24 (80%)		15 (71.4%)	49 (76.6%)		28 (73.7%)	36 (76.6%)		29 (87.9%)	35 (67.3%)	

**Table 3:** Correlation between MetS components and coffee consumption amount (N = 85).

Variable	TGs		HDL		FBG		WC		SBP		DBP	
	r *	*p*-Value	r	*p*-Value	r	*p*-Value	r	*p*-Value	r	*p*-Value	r	*p*-Value
Coffee cup size (mL)	−0.020	0.855	0.122	0.275	0.104	0.322	0.299	0.038	0.033	0.765	−0.034	0.757
Coffee cups/day	0.164	0.134	0.050	0.655	0.119	0.279	−0.078	0.486	0.045	0.681	−0.119	0.279

* r: correlation coefficient.

**Table 4:** Relationship between coffee consumption and BMI, TC, and LDL (N = 85).

		BMI		LDL		TC	
		Mean ± SD	*p*-Value	Mean ± SD	*p*-Value	Mean ± SD	*p*-Value
Coffee cup size	S	33.3 ± 7.6	0.133	3.0 ± 1.0	0.578	4.7 ± 1.2	0.498
	M	32.9 ± 5.3		2.8 ± 0.9		4.4 ± 1.0	
	L	35.0 ± 5.5		3.2 ± 1.1		5.0 ± 1.2	
	XL	37.1 ± 6.0		3.2 ± 0.8		4.9 ± 1.0	
Coffee cups/day	<1 (once a week)	43.1 ± 11.5	0.008	4.0 ± 1.6	0.052	5.7 ± 1.7	0.046
	1	32.6 ± 4.5		2.8 ± 0.8		4.4 ± 1.1	
	2	33.9 ± 5.8		3.2 ± 0.9		4.9 ± 0.9	
	3	34.0 ± 4.8		2.8 ± 0.7		4.4 ± 1.0	
	>3	35.1 ± 6.5		3.2 ± 1.0		4.9 ± 1.1	
Types of coffee	Arabic	34.8 ± 6.6	0.407	3.1 ± 1.0	0.668	4.7 ± 1.1	0.469
	Cappuccino	33.9 ± 6.2		3.3 ± 0.8		4.9 ± 0.9	
	Black	35.0 ± 7.6		3.2 ± 1.1		4.8 ± 1.4	
	Turkish	32.7 ± 7.1		2.8 ± 1.0		4.2 ± 1.2	
	Instant	37.8 ± 0.9		3.0 ± 1.0		4.5 ± 1.1	
	Americano	25.2 ± 0.0		1.2 ± 0.0		2.2 ± 0.0	
	Cold	43.2 ± 0.0		2.9 ± 0.0		4.1 ± 0.0	
	Latte	30.0 ± 0.0		3.7 ± 0.0		5.1 ± 0.0	
	Mocha	34.4 ± 6.6		2.9 ± 0.1		4.7 ± 0.1	
Coffee additives	Milk	35.6 ± 6.4	0.515	3.3 ± 0.7	0.655	5.1 ± 0.9	0.704
	Sugar	36.0 ± 5.1		3.1 ± 0.4		4.5 ± 0.8	
	Sweetener	33.9 ± 10		3.1 ± 1.3		4.8 ± 1.3	
	Syrup	45.0 ± 0.0		4.5 ± 0.0		6.3 ± 0.0	
	Shredded chocolate	37 ± 0.0		3.7 ± 0.0		4.7 ± 0.0	
	Stevia sugar	40.6 ± 4.9		2.8 ± 0.9		4.5 ± 1.0	
	None	34.0 ± 6.9		3.0 ± 1.0		4.7 ± 1.2	

**Table 5:** Correlation between MetS components and coffee consumption amount (N = 85).

Variable	BMI		LDL		TC	
	r *	*p*-Value	r	*p*-Value	r	*p*-Value
Coffee cup size (mL)	0.282	0.009	0.079	0.486	0.104	0.357
Coffee cups/day	−0.015	0.891	0.011	0.920	−0.008	0.946

* r: correlation coefficient.

**Table 6:** Binary logistic regression analysis (inter-method) for predictors of high FBG.

		β *	95% CI		*p*-Value
			Lower	Upper	
Coffee cup size		0.842	0.559	1.269	0.412
Number of coffee cups/day		0.691	0.468	1.019	0.062
Types of coffee	Arabic	2.143	0.531	8.646	0.284
	Cappuccino	1.517	0.427	5.395	0.519
	Black	0.452	0.178	1.150	0.096
	Turkish	1.304	0.303	5.618	0.721
	Instant	2.381	0.603	9.359	0.215
	Americano	--	0.0	0.0	1.00
	Cold	--	0.0	0.0	1.00
	Latte	--	0.0	0.0	1.00
	Mocha	0.627	0.038	10.389	0.745
Coffee additives	Milk	4.159	0.859	20.133	0.077
	Sugar	--	0.0	0.0	0.999
	Sweetener	1.280	0.111	14.702	0.843
	Syrup	--	0.0	0.0	1.00
	Shredded chocolate	--	0.0	0.0	1.00
	Stevia sugar	0.627	0.038	10.389	0.745
	None	0.284	0.086	0.938	0.039

* β = odds ratio; CI = confidence interval.

## Data Availability

Data are available on request from the authors.

## Abbreviations

The following abbreviations are used in this manuscript:

HDL-C	High-density lipoprotein cholesterol
LDL-C	Low-density lipoprotein cholesterol
TC	Total cholesterol
TG	Triglyceride
HbA1c	Hemoglobin A1c
FBG	Fasting blood glucose
WC	Waist circumference
BP	Blood glucose
